# The Death of Dr. Barry

**Published:** 1897-10-16

**Authors:** 


### The Death of Dr. Barry.

The death of Dr. F. W. Barry, Senior Medical Inspector to the Local Government Board, cannot be allowed to pass without an expression of regret at the sudden termination of a career so useful and so honourable as his has been. The public owes to Dr. Barry more than it knows, or probably ever will know, for the work at the Medical Department only occasionally comes within the ken of the outside world. Among other duties, it fell to his lot to keep a constant watch on the progress of cholera during those years when that disease ran over Europe, and again and again threatened England. He also was engaged in making that sanitary survey of our coast-line which aided so greatly in enabling proper precautions to be taken to prevent the admission of the disease. His report also on cholera in England in 1893, and that on the western diffusion of cholera during the year 1892, are admirable documents; but there is no doubt that Dr. Barry will chiefly be remembered, so far as his public life is concerned, by his masterly researches into the cause of the prevalence of enteric fever in the Tees Valley, and by his "Report on an Epidemic of Small-pox at Sheffield." Both these reports drew upon him much public attention, besides no small abuse from his opponents. In these investigations he spared neither time nor labour, and he produced in each case reports which are monuments of careful observation and of logical deduction—sufficient to make a high reputation for any man. We cannot but feel that in both cases he demonstrated the truth, and that in so forcible a manner as to give a permanent impress to the sanitary progress of his country. His death is a great loss.

